# Portable Electric Lamps

**Published:** 1908-10-24

**Authors:** 


### PORTABLE ELECTRIC LAMPS.

THE portable electric lamps arranged for surgeons' use all consist of a case containing a battery, which may be of the primary or secondary type, with a small electric incandescent lamp attached, and with some arrangement, such as a spring, for connecting the battery to the lamp when the surgeon requires it. Where the batteries are of the primary type, they consist of dry cells made in the form of a cartridge, which can be slipped into the case when the previous one is exhausted. Primary batteries are not very much used for the purpose, because it is difficult to get a sufficient quantity of the active materials required within the space that can be allowed. Where, however, there is no charging current within convenient reach, the primary battery will answer the purpose, providing it is carefully watched. It should be tested fairly frequently, and always before use, and if the light has decreased appreciably a new battery should be put in.

The secondary batteries usually form part of the case itself. They are small editions of the batteries that are employed in electricity generating stations, in which lead plates are used, and they require to be charged by a current of electricity before they will furnish the required current for the lamp. They may be charged from any electricity supply, whether continuous current or alternating; but it is necessary, when arranging to charge them, to know whether the current is continuous or alternating. The alternating current is that which surgeons know as the sinusoidal.

Where the electricity supply is on the continuous, or, as it is sometimes called, the direct current system, the charging is done by taking connections from, say, two wires that are arranged to supply current to one of the lamps of the house, and forming a circuit with them the battery to be charged and an electric incandescent lamp. The c.p. of the electric incandescent lamp to be used depends upon the size of the battery, but for the small portable lamps an 8 c.p. lamp will usually be sufficient. It must be a lamp arranged to work with the full pressure of the service, and it is necessary to include it in the circuit with the battery, in order to reduce the current passing through the battery to the proper figure. Surgeons who are using portable lamps regularly will be wise to have a little charging board fixed in some convenient place.

With alternating currents an apparatus called a rectifier, supplied by firms who make surgical appliances, must be included in the circuit with the charging battery.

Secondary batteries should never be allowed to run down. It may be taken as a good rule to give them a charge always after using the lamp; and, further, if the lamp is laid aside for a time out of use, it should be frequently tested, and frequently charged. Immediately the light shows any diminution the battery should be given a fresh charge. The small batteries used with portable lamps particularly require this attention. Secondary chemical actions go on within the battery, if it is not constantly fed with current, which gradually deteriorate the plates, and render the battery useless.

Another important point is the switch. Exigencies of space only allow of very small pieces of metal for this purpose, and with continual use the contacts are apt to get dirty; and, in addition, the springs of which the switch is formed are apt to take a permanent set, either just in contact or just not making contact when required. The surgeon who uses these batteries will be wise to make himself master of the switch.

Secondary batteries must be charged for eight to ten hours, when much run down, and for an hour or two hours if they have only just commenced to lose power. The connecting wires or springs between the battery and the lamp should also be examined frequently. If acid spills over them they are quickly eaten away.

